# Interoception is associated with anxiety and depression in pregnant women: A pilot study

**DOI:** 10.1371/journal.pone.0267507

**Published:** 2022-05-06

**Authors:** Minami Noda, Yoko Sato, Yoshiko Suetsugu, Seiichi Morokuma

**Affiliations:** Department of Health Sciences, Graduate School of Medical Sciences, Kyushu University, Fukuoka, Japan; Dartmouth College Geisel School of Medicine, UNITED STATES

## Abstract

Pregnancy and postpartum are periods in which women develop psychosocially. However, becoming a mother is stressful, and mood disorders related to anxiety and depression often develop. In recent years, research on interoception—sensations related to the body’s internal physiological state—has attracted attention. Interoception has multifaceted characteristics. It involves directly perceiving information in the body while also inferring and evaluating it. In this study, we examined interoception, anxiety, and depression in Japanese pregnant women. Empirical examinations and questionnaire surveys were used to measure interoception in 32 pregnant women not at high risk of pregnancy. A Japanese adaption of the Multidimensional Assessment of Interoceptive Awareness was used to measure interoceptive sensibility, and a heartbeat counting task performance was used to measure interoceptive accuracy. Anxiety and depression were measured using the Japanese versions of the State-Trait Anxiety Inventory and the Edinburgh Postnatal Depression Scale, respectively. A correlation analysis was performed between interoception, anxiety and depression and between differences between sensibility and accuracy of interoception, anxiety and depression. We revealed that interoceptive sensibility and differences between sensibility and accuracy of interoception were associated with anxiety. Based on results of this pilot study, it is necessary to investigate using longitudinal studies whether interoception might be an effective predictor tool for early detection of anxiety during pregnancy and postpartum.

## Introduction

During pregnancy and the postpartum period, mood disorders related to anxiety and depression often develop. The incidence of depression during pregnancy in Japan is 5.6% [1], and that of postpartum depression is as high as 10–20% [2]. Since it is necessary to grasp mothers’ mental state, Cox and colleagues developed the Edinburgh Postnatal Depression Scale (EPDS), a postnatal depression screening test in the United Kingdom [3].

A previous study that used the EPDS to examine the relationship between depression during pregnancy and postpartum depression showed that those with a high EPDS score during pregnancy had significantly higher scores after birth [4], suggesting that postpartum depression is associated with depression during pregnancy. Furthermore, when anxiety during pregnancy was evaluated using the State-Trait Anxiety Inventory (STAI), a significant correlation with postpartum depression was reported [5]. In addition, as a result of meta-analysis, it has been reported that depression during pregnancy is also associated with preterm birth and low birth weight infant birth [6].

From the above, to prevent postpartum depression and other complications, interventions should be developed for pregnant women prone to depression and anxiety from the beginning of pregnancy. However, questionnaire surveys about mental health show that some pregnant women tend to avoid expressing their emotions [4].

In recent years, research on the mind—body correlation or “interoception”—a sensation related to the physiological state inside the body—has attracted attention. The term interoception was coined by Sherrington [7] and is intended to represent conscious awareness of the homeostasis of the entire body. In addition, there are individual differences in interoception, which are important sensations related to one’s own feelings, consciousness, and self-awareness [8].

Interoception consists of the function of directly perceiving information in the body and the cognitive function of inferring and evaluating [9]. To measure interoceptive sensibility, a questionnaire is used to evaluate individuals’ internal state and the tendency to pay attention to oneself, and a heartbeat counting task is performed to measure interoceptive accuracy as a function of perception [10]. It is also said that deviations between sensibility and accuracy of interoception can lead to mental and physical illnesses such as mood disorders and metabolic disorders [11].

Studies concerning interoception in pregnant women are scarce. In a research on interoception in pregnant women, researchers considered mindfulness; i.e., “paying attention in a particular way: on purpose, in the present moment, and non-judgmentally” [12]. In their study, they focused on primiparas with fear and anxiety about childbirth and provided them with a mindfulness-based preparatory education for childbirth (i.e., “Mind in Labor”) in the second half of their pregnancy, just before childbirth, and also within six weeks after childbirth. They examined the psychological effects of the Mind in Labor intervention over time [13]. It was reported that pregnant women who received the Mind in Labor education showed reduced postpartum depressive symptoms and increased interoception scores as compared to pregnant women who received the standard treatment [13]. Thus, although reports show the effectiveness of mindfulness, there are no reports that examine the relationship between pregnant women’s interoception, anxiety, and depression during pregnancy. Therefore we first aimed to clarify the relationship between interoception (sensibility and accuracy), anxiety, and depressive tendencies in pregnant women.

## Materials and methods

### Participants

This study was approved by the ethics committee of Kyushu University Hospital (no. 2019–121), and all mothers who participated in the study provided written informed consent prior to study commencement. All study procedures were performed following the tenets of the Declaration of Helsinki.

We performed a cross-sectional study with 32 pregnant women aged 20 years and older from 22 to 29 weeks of gestation of their pregnancy with stable hemodynamics, from the obstetric outpatient department of the Kyushu University Hospital. The study was conducted from July to September 2019. Mothers with apparent fetal morphological abnormalities or maternal complications at the time of recruitment were excluded.

### Materials

#### Basic information

We obtained mothers’ health status and personal data (e.g., age, weeks of pregnancy, educational background, past medical history, current medical history, obstetrical history, height, weight, drinking status, smoking status, infertility treatment [this time], employment status, and financial status) from their medical records and questionnaires.

#### Anxiety

We used the Japanese version of the STAI to determine mothers’ anxiety levels. The STAI was created by Spielberger [14], and the Japanese version was adapted by Shimizu and colleagues [15]. Although this scale includes a state and a trait scale, in this study, we only used the trait scale.

#### Depression

We used the EPDS to measure depression. The EPDS was created by Cox and colleagues for quantitatively evaluating postnatal maternal depression [3], and the Japanese version was adapted by Yamashita and colleagues [16]. At present, it is also used to investigate depression status during pregnancy and has confirmed validity for the pregnancy period [17].

#### Subjective measure of interoception (sensibility of interoception)

We used the Multidimensional Assessment of Interoceptive Awareness (MAIA), developed by Mehling and colleagues, to measure subjective interoception [18]. Mehling and colleagues and Shoko and colleagues jointly conducted a factor analysis and created the Japanese version of the MAIA (MAIA-J) [19]. The Japanese version includes six subscales: “Noticing”,“Not-distracting”,“Attention regulation”, “Emotional awareness”,“Body listening”, and “Trusting”.

#### Objective measure of interoception (accuracy of interoception)

The objective measurement of interoception was based on the method by Schandry using heart beat counting task performance procedure [10]. Participants in the 25-, 35-, and 45-second segments count their own heartbeat without cues and record it after each segment. The participant-reported heart rate and the actual heart rate measured from the electrocardiogram in each of the three segments are calculated and compared. The absolute value of the difference between the reported heart rate and the actual heart rate is calculated for each of the three sections, and this value is divided by the actual heart rate to calculate the ratio of the heart rate deviation [8, 10].

### Procedure

The study was conducted in a quiet outpatient private room to avoid outside noise. First, a wearable heart rate sensor (WHS-1, Union Tool Co., Japan) was attached to the left precordial area and the participants were allowed to sit and rest for five minutes. Then, the heartbeat counting task was conducted. Lastly, participants completed the questionnaires (EPDS and MAIA), which were collected from them immediately after.

### Analytical methods

First, descriptive statistics were calculated. The MAIA-J, heart ratebeat task performance, STAI, and EPDS scores were classified into two groups: age (≤ 34 or ≥ 35), parity (primipara or multiparous), and gestational weeks (22–25 or 26–29). The Mann-Whitney U test was used to determine whether there were significant differences in MAIA-J, heartbeat task performance, STAI, and EPDS scores. Subsequently, Spearman’s rank correlations were calculated for heartbeat counting task performance, MAIA-J, STAI, and EPDS (total score). All analyses were conducted with IBM SPSS ver. 25 (SPSS; IBM, Armonk, NY, USA), and significance was set at 5%.

## Results

Participants’ basic and clinical characteristics are shown in Table 1.

**Table 1:** Participants’ Characteristics. (n = 32).

Item	Mean ± SD or n (%)
Mothers’ age (years)	32.8 ± 4.9 (range = 25–42)
Parity	
Primipara	16 (50.0%)
Multiparous	16 (50.0%)
Gestational weeks	26 ± 2.1 (range = 22–29)
Body mass index (before pregnancy)(kg/m^2)	
Underweight (<18.5)	4 (12.5%)
Normal weight (18.5–25)	24 (75.0%)
Overweight (25≧)	4 (12.5%)
Alcohol Drinking	
No alcohol drinking	25 (78.1%)
Sometimes	7 (21.9%)
Smoking	
No smoking	29 (90.6%)
Previously smoked	3 (9.4%)
Fertility treatments during this pregnancy	
No	24 (75.0%)
Yes	8 (25.0%)
Employment status	
Not working	14 (43.8%)
Working	18 (56.3%)
Educational background	
High school	6 (18.8%)
Junior college/vocational school	9 (28.1%)
University/graduate school	17 (53.1%)
Financial anxiety	
Not at all	0 (0%)
Slightly	16 (50.0%)
Not sure	5 (15.6%)
Never thought about it	10 (31.3%)
Mostly	1 (3.1%)

The descriptive statistics for each study scale used are shown in Table 2.

**Table 2:** Descriptive Statistics for each scale.

Scale		Mean ± SD	Median	IOR^d^Min–Max
MAIA^a^	Noticing	2.9 ± 0.9	3.00	2.40–3.40
	Not-distracting	2.61 ± 1.2	2.33	1.67–3.67
	Attention regulation	2.70 ± 0.7	2.71	2.25–3.14
	Emotional awareness	3.33 ± 1.1	3.67	2.67–4.00
	Body listening	2.86 ± 1.0	2.88	2.25–3.50
	Trusting	3.07 ± 0.9	3.17	2.67–3.67
Heartbeat counting task performance		0.62 ± 0.2	0.61	0.54–0.74
STAI^b^ score		45.8 ± 5.2	46	40.75–49.50
EPDS^c^ score		5.47 ± 3.0	5	3–7

^a^ Multidimensional Assessment of Interoceptive Awareness

^b^ State Trait Anxiety Inventory

^c^ Edinburgh Postnatal Depression Scale

^d^ Interquartile Range.

### Comparison of interoception across participants’ characteristics

As the result of comparison of interoception across participants’ characteristics, there was a significant difference between primipara and multiparous women in MAIA-J subscale “Attention regulation” (Table 3). “Attention regulation” of primipara women was significantly lower (*p* = .003) than those of multiparous women (median score±SD 2.34 ±0.6 compared with 3.06 ±0.6, *p* = .003, Mann Whitney U test). There was no significant difference in MAIA-J scores and heartbeat counting task performance across ages (≤ 34 and ≥ 35) and gestational weeks (22–25 weeks and 26–29 weeks).

**Table 3:** Comparison of interoception by participants’ parity.

Item	n (%)	MAIA-J^a^ (Mean ± SD)												Performance^d^	p
		Noticing	p^c^	Not-distracting	p	Attention regulation	p	Emotional awareness	p	Body listening	p	Trusting	p		
Primipara	16 (50.0%)	3.03 ± 1.0	N.S.	2.54 ± 1.1	N.S.	2.34 ± 0.6	0.003	3.17 ± 1.2	N.S.	3.00 ± 1.1	N.S.	2.88 ± 1.0	N.S.	0.60 ± 0.1	N.S.
Multiparous	16 (50.0%)	2.78 ± 0.7		2.69 ± 1.4		3.06 ± 0.6		3.50 ± 1.0		2.77 ± 1.0		3.27 ± 0.9		0.64 ± 0.1	

^a^ MAIA-J = Japanese version of the Multidimensional Assessment of Interoceptive Awareness;

^b^ N.S. = not significant.

^c^ Mann-Whitney U test. p < .05.

^d^ Performance = heartbeat counting task performance

### Correlations between interoception (sensibility and accuracy), anxiety, and depression

The correlations between interoception (sensitivity and accuracy) and anxiety and depression are shown in Table 4 with spearman correlation. There was a significant positive correlation between “Not-distracting” and the STAI scores (*ρ* = 0.452, *p* < .01), a significant negative correlation between “Attention regulation” and the STAI scores (*ρ* = -0.492, *p* < .01), and a significant positive correlation (*ρ* = 0.455, *p* < .01) between “Body listening” and the depressive factor. Further, there were significant negative correlations between “Emotional awareness” and heartbeat counting task performance (*ρ* = -0.441, *p* < .05), “Body listening” and heartbeat counting task performance (*ρ* = -0.499, *p* < .01), and “Trusting” and heartbeat counting task performance (*ρ* = -0.477, *p* < .01).

**Table 4:** Spearman correlations between interoception (sensibility and accuracy), anxiety, and depression.

		①	②	③	④	⑤	⑥	⑦	⑧	⑨
MAIA-J	① Noticing	1								
	② Not-distracting	-.071	1							
	③ Attention regulation	.066	.229	1						
	④ Emotional awareness	.721**	.048	.139	1					
	⑤ Body listening	.520**	.024	.076	.533**	1				
	⑥ Trusting	.418*	-.004	.189	.470**	.495**	1			
	⑦ Heartbeat counting task performance	-.315	-.244	-.210	-.441*	-.499**	-.477**	1		
	⑧ STAI score	.452**	-.165	-.492**	.329	.300	.175	-.102	1	
	⑨ EPDS score	.204	-.197	-.043	-.042	.331	.092	-.107	.168	1

### Correlation between the difference in sensibility and accuracy of interoception and anxiety and depression

Table 5 shows correlations between the difference in sensibility and accuracy of interocception and anxiety and depression. The difference between “Noticing” and heartbeat counting task performance showed a significant positive correlation with STAI (ρ = 0.387, p < .05).

**Table 5:** Correlation between the difference in sensibility and accuracy of interocception and anxiety and depression.

		①	②	③	④	⑤	⑥	⑦	⑧	⑨	⑩	⑪
MAIA-J—performance*	① Noticing—performance*	1										
	② Not-distracting—performance	.583**	1									
	③ Attention—performance	.612**	.656**	1								
	④ Emotional awareness- performance	.897**	.611**	.651**	1							
	⑤ Body listening—performance	.842**	.646**	.636**	.832**	1						
	⑥ Trusting—performance	.790**	.620**	.748**	.791**	.832**	1					
	⑦ STAI score	**.387** *	-.040	-.202	.269	.259	.123	1				
	⑧ EPDS score	.191	-.026	.072	.042	.247	.083	.168	1			

From ①—⑥, data are the difference between MAIA subscale and heartbeat counting task performance.

*performance = heartbeat counting task performance.

## Discussion

We found that interoception sensibility and the difference between sensibility and accuracy of interoception in pregnant women were associated with anxiety and depression through this research.

### Relationship between interoception sensibility and anxiety in pregnant women

Those with low “Attention regulation” tended to have high anxiety, as measured by the STAI. Since the gray matter volume of the prefrontal cortex, which controls attention control function, decreases during pregnancy [20], we infer that some pregnant women tend to have low attention regulation. In addition, it was reported that in patients with anxiety disorders, there is a decrease in the ability to control attention [21]. That is, in pregnant women, reduced ability to sustain and control attention to body sensations may be related to anxiety. Furthermore, there were differences between primiparas and multiparous women regarding the scores of “Attention regulation” and anxiety. Namely, primipara group tended to have lower attention regulation and higher anxiety. Regarding anxiety, Iwata and colleagues showed that primiparas had higher trait anxiety than multiparous [22], supporting the current findings. Primiparas face unprecedented realities such as physical and psychological changes associated with pregnancy and becoming a mother.

It was also found that those who have high “Noticing” also tended to have high anxiety. According to Terasawa and colleagues, people with a keen sense of interoception noticing tend to have high overt anxiety [23, 24]. “Noticing” can be an indicator of anxiety.

### Relationship between interoception sensibility and accuracy in pregnant women

Those with a large dissociation between “Noticing” and heartbeat counting task performance tended to have high anxiety. A study of the relationship between interoception and anxiety reported that the group with high performance on the heartbeat perception task had low speech and health anxiety [25].

The insular cortex plays an important role in the neural basis of interoception, which is based on experience [26]. When the discrepancy between objective interoception and subjective interoception is small, interoception is considered to be appropriate [11, 27]. However, when the discrepancy is large, interoception is considered inappropriate, and can cause mental and physical problems such as mood and metabolic disorders [11]. Endocrine functional changes caused by hypothalamic overactivity and inflammatory conditions may deviate from the prediction of the insular cortex and increase prediction error [28]. We considered that pregnant women are in a state where it is difficult to maintain an appropriate interoception as the perceptive abilities of the brain are not on adequate levels owing to external changes in the body that occur daily, and the internal changes caused by rapid hormonal dynamics [20, 27, 29]. Therefore, we should recognize that pregnant women may have difficulty maintaining an interoception.

### Future prospects

Unlike the EPDS and STAI, the MAIA-J does not directly measure respondents’ mental health; thus, it could effectively be used to capture the mental aspect. Based on the results of this pilot study, it is necessary to investigate whether interoception can be an effective screening tool for early detection of anxiety during pregnancy and postpartum through longitudinal studies.

### Limitations

The target facility was a university hospital and the sample size was small; therefore, there are limits to the generalizability of our findings. Participants’ pregnancy duration ranged from 22 to 29 weeks, and it is difficult to control the internal changes associated with pregnancy in this period. Therefore, the study participants’ performance in the heartbeat counting task may have been negatively affected. Since all participants were pregnant women, the results need to be compared to those of non-pregnant women. Lastly, since this was a correlational study, the sample size should be increased and an experimental design should be employed to evaluate possible causal relationships between these variables.

## Conclusion

We present a novel finding that interoception sensibility and the difference between interoception sensibility and interoception accuracy were associated with anxiety.

## Supporting information

S1 File (PDF)
