# Supplementary material for: Interoception is associated with anxiety and depression in pregnant women: A pilot study
Source: PLoS One. 2022 May 6;17(5):e0267507. doi: 10.1371/journal.pone.0267507 (PMC9075621; doi:10.1371/journal.pone.0267507)
Supplement: S1 File — (PDF) [file pone.0267507.s001.pdf]

No	Age	Parity	Gestational weeks	BMI	Alcohol Drinking	Smoking	Fertility treatments during this pregnancy	Employment status	Educational background	Financial anxiety	STAI trait	EPDS	MAIA-J	Noticing	Not-distracting	Attention regulation	Emotional awareness	Body listening	Trusting	heart beat counting
A001	25	G1P0	25	33.30	1	0	0	1	3	2	51	8	109	4.60	1.00	2.71	4.33	4.50	3.33	0.3
A002	26	G2P1	26	18.82	1	0	1	1	4	4	39	4	82	1.80	2.33	3.71	2.00	1.00	3.67	0.6
A003	28	G1P0	27	18.29	0	0	0	0	4	2	44	5	78	2.60	1.67	2.14	2.67	2.25	4.00	0.7
A004	32	G2P0	28	27.41	0	0	0	1	3	2	47	3	57	0.80	1.67	1.43	2.00	3.50	1.33	0.7
A005	33	G1P0	28	20.20	0	0	1	0	2	4	38	4	71	1.60	4.33	2.43	1.00	2.00	3.00	0.5
A006	31	G1P0	27	23.73	0	1	1	1	3	2	46	2	85	3.60	1.67	2.57	4.00	2.75	3.00	0.5
A007	32	G1P0	25	21.36	0	0	0	0	2	2	46	6	78	3.20	4.33	2.57	2.67	1.75	1.33	0.8
A008	32	G3P2	25	24.34	0	0	1	0	4	4	41	3	104	3.00	5.00	3.00	3.67	3.00	3.33	0.4
A009	42	G10P4	29	19.53	0	0	0	1	4	4	39	4	59	1.00	1.67	3.29	1.00	1.00	1.67	0.8
A010	26	G1P0	25	21.87	0	0	0	1	4	2	52	6	99	3.20	2.33	2.57	4.33	3.50	3.67	0.4
A011	34	G3P0	29	22.37	1	0	1	0	4	4	53	11	89	3.20	2.67	2.29	4.00	3.00	2.00	0.6
A012	37	G2P1	28	19.98	0	0	0	1	3	3	46	6	114	3.00	2.67	3.71	4.00	4.25	5.00	0.6
A013	29	G1P0	23	22.23	0	0	0	1	4	2	45	3	88	3.40	2.33	3.00	3.67	1.75	2.33	0.6
A014	36	G2P0	29	27.89	0	0	0	0	4	4	57	5	42	2.00	1.00	1.29	0.67	0.50	1.00	0.9
A015	36	G2P0	26	23.94	0	0	1	0	4	5	51	7	107	4.00	3.33	1.86	5.00	4.25	4.00	0.4
A016	36	G2P1	26	18.31	0	1	0	1	2	2	39	6	97	3.00	1.00	3.71	3.67	3.00	3.33	0.6
A017	39	G2P1	24	18.82	0	0	0	1	4	3	40	3	101	3.40	3.33	3.14	4.00	2.25	2.33	0.8
A018	32	G2P1	27	23.78	0	0	0	1	3	2	43	5	83	2.40	2.33	2.86	2.67	2.75	3.00	0.7
A019	35	G2P0	25	22.21	0	0	1	1	4	2	52	15	82	3.40	2.00	2.00	3.00	3.50	3.00	0.6
A020	37	G3P0	29	22.03	0	0	1	1	3	2	40	9	97	2.20	3.67	3.29	2.67	3.25	3.00	0.5
A021	31	G2P1	22	18.37	0	0	0	1	4	3	40	7	82	2.60	1.67	2.71	3.00	2.75	2.67	0.8
A022	27	G2P1	23	20.00	0	0	0	1	3	2	46	7	86	3.00	1.33	2.00	3.33	4.00	3.33	0.7
A023	25	G2P1	27	23.23	0	0	0	0	3	4	52	3	85	3.20	0.33	1.71	5.00	2.25	3.67	0.6
A024	34	G1P0	28	17.90	1	0	0	0	4	4	49	8	112	3.80	3.67	3.14	3.33	4.00	3.67	0.5
A025	41	G2P1	23	22.77	1	0	0	0	4	3	37	4	99	2.40	4.67	2.86	4.00	3.25	2.67	0.5
A026	27	G3P2	27	26.45	0	0	0	0	2	4	53	0	128	3.80	4.00	3.71	4.33	4.50	5.00	0.5
A027	32	G2P0	25	21.60	1	0	0	1	4	2	45	4	120	4.00	2.33	2.71	4.67	4.50	4.00	0.7
A028	31	G3P1	29	19.53	1	0	0	1	4	4	48	3	92	2.60	3.67	2.57	3.00	2.50	3.00	1.0
A029	30	G2P1	23	19.74	0	1	0	0	2	2	44	10	101	3.80	1.33	4.00	4.00	3.00	3.33	0.7
A030	42	G1P0	25	19.23	0	0	0	1	2	2	48	5	77	2.80	2.67	1.43	2.67	2.25	3.33	0.8
A031	41	G3P1	25	19.30	0	0	0	0	3	2	48	6	96	3.00	3.33	2.86	4.00	2.50	3.67	0.4
A032	32	G3P1	25	21.64	0	0	0	0	4	3	45	3	94	2.40	4.33	3.14	4.33	2.25	2.67	0.6
